# Circular RNA Expression for Dilated Cardiomyopathy in Hearts and Pluripotent Stem Cell–Derived Cardiomyocytes

**DOI:** 10.3389/fcell.2021.760515

**Published:** 2021-12-17

**Authors:** Yiyu Zhang, Guoqing Huang, Zhaohu Yuan, Yonggang Zhang, Rong Chang

**Affiliations:** ^1^ Department of Blood Transfusion, Department of Cardiology, Shenzhen Longhua District Central Hospital, The Affiliated Central Hospital of Shenzhen Longhua District, Guangdong Medical University, Shenzhen, China; ^2^ Department of Blood Transfusion, Guangzhou First People’s Hospital, School of Medicine, South China University of Technology, Guangzhou, China

**Keywords:** circular RNAs, heart diseases, arrhythmia, hiPSC disease modeling, dilated cardiomyopathy

## Abstract

Dilated cardiomyopathy (DCM) is a type of heart disease delimited by enlargement and dilation of one or both of the ventricles along with damaged contractility, which is often accompanied by the left ventricular ejection fraction (LVEF) less than 40%. DCM is progressive and always leads to heart failure. Circular RNAs (circRNAs) are unique species of noncoding RNAs featuring high cell-type specificity and long-lasting conservation, which normally are involved in the regulation of heart failure and DCM recently. So far, a landscape of various single gene or polygene mutations, which can cause complex human cardiac disorders, has been investigated by human-induced pluripotent stem cell (hiPSC) technology. Furthermore, DCM has been modeled as well, providing new perspectives on the disease study at a cellular level. In addition, current genome editing methods can not only repair defects of some genes, but also rescue the disease phenotype in patient-derived iPSCs, even introduce pathological-related mutations into wild-type strains. In this review, we gather up the aspects of the circRNA expression and mechanism in the DCM disease scenario, facilitating understanding in DCM development and pathophysiology in the molecular level. Also, we offer an update on the most relevant scientific progress in iPSC modeling of gene mutation–induced DCM.

## Introduction

Dilated cardiomyopathy (DCM) is genetically and phenotypically heterogenous, accompanied by left ventricular dilatation and dysfunction. It is the most common form of cardiomyopathy in both adults and children in the world. DCM is non-ischemic and progressive, with an increased risk of heart failure (Jefferies and Towbin, 2010). The continuous expansion of the ventricle leads to a decline in left ventricular ejection fraction (LVEF), which in turn leads to abnormalities in the extra myocardial matrix, ventricular arrhythmia, and heart failure. It has been assumed to cause diseases such as viral myocarditis and other rheumatological diseases, while endocrinological disorders might further contribute to occurrence of DCM (Hänselmann et al., 2020; Imanaka-Yoshida, 2020). For example, COVID-19 patients hold higher risk of developing DCM due to continuous immune activation (Komiyama et al., 2020). Importantly, the occurrence of heart failure and arrhythmia determines patient’s poor or good prognosis. Most DCM patients need transplantation to increase the survival rate.

In past several years, the study of circRNAs has opened a new avenue for survey in DCM research (Lei et al., 2018). Increasing evidences show that circRNAs have dynamic changes and tissue specificity in several different cardiovascular diseases, which are derived from DCM (Holdt et al., 2018). While DCM development is progressive, circRNAs can be used as biomarkers for DCM disease diagnosis and therapeutic targets for the single gene mutation in DCM treatment (Lei et al., 2018). Importantly, the study of function of circRNAs offers a valuable resource that can be used to further explore the diagnostic standard and treatment of DCM for tissue specificity genes in heart diseases. Because the development of disease is a long and gradual process, it is convenient to use human cells or tissue disease models as platforms for performing observation and research (Lei et al., 2018).

In this review, we not only discuss the research on mechanisms and roles of circRNAs in DCM but also talk about the hiPSC modeling method for DCM disease investigation with the function and mechanism of circRNAs.

### What is DCM?

Heart failure is generally caused by either DCM or ischemic cardiomyopathy (ICM). DCM, a type of cardiomyopathy, is the leading cause of heart transplantation. It occurs most likely in young population with high mortality–morbidity risk, which is attributed to a combination of genetic and acquired triggers. So far, the clinical measure of DCM is mainly based on ejection fraction (EF) and NYHA systematization, without considering the heterogeneity of DCM (Priori et al., 2015; Ponikowski et al., 2016). Overall, nearly 40–50% of DCM patients can relieve from heart failure therapy, with a genetic basis (Merlo et al., 2011; Verdonschot et al., 2018). The clinical symptoms of DCM in children are different from those of adults in some places, such as coarse faces and slightly dilated heart (Carboni et al., 2020). Histological examination of DCM hearts shows evidence of nonspecific changes as well as myocardial hypertrophy and fibrosis (Bakalakos et al., 2018). Biopsy exposes may put patients under unnecessary risk because of idiopathic DCM being nonspecific (Palomer et al., 2018). Familial forms occupy the 40% of cases, but many pathogenic genes are irregular, intergenerational inheritance.

### What is DCM-Related Gene?

While studying the role of circRNAs in the development of DCM diseases, we should primarily consider some genes related to the development of DCM disease. It is because most circRNAs are classic noncoding RNA molecules, while only a little of ribosome-associated circRNAs can produce detectable peptides (van Heesch et al., 2019). The function of circRNA has been widely confirmed as an essential role for miRNA sponges to affect mRNA expression to regulate the synthesis of disease-related proteins and/or influence its parental gene expression to produce the protein to affect the biological progress of the disease. Whether and how these circRNAs are relevant to other forms of the mechanism to regulate DCM-related mRNA remains to be studied.

Adverse consequences of DCM generally lead to heart failure, arrhythmias, and sudden cardiac death, and the fundamental reasons are attributed to genetic and environmental factors. In the past few decades, single mutations in genes encoding muscle fibers, cytoskeleton, and channel proteins have been found to be associated with DCM.

**FIGURE 1:**
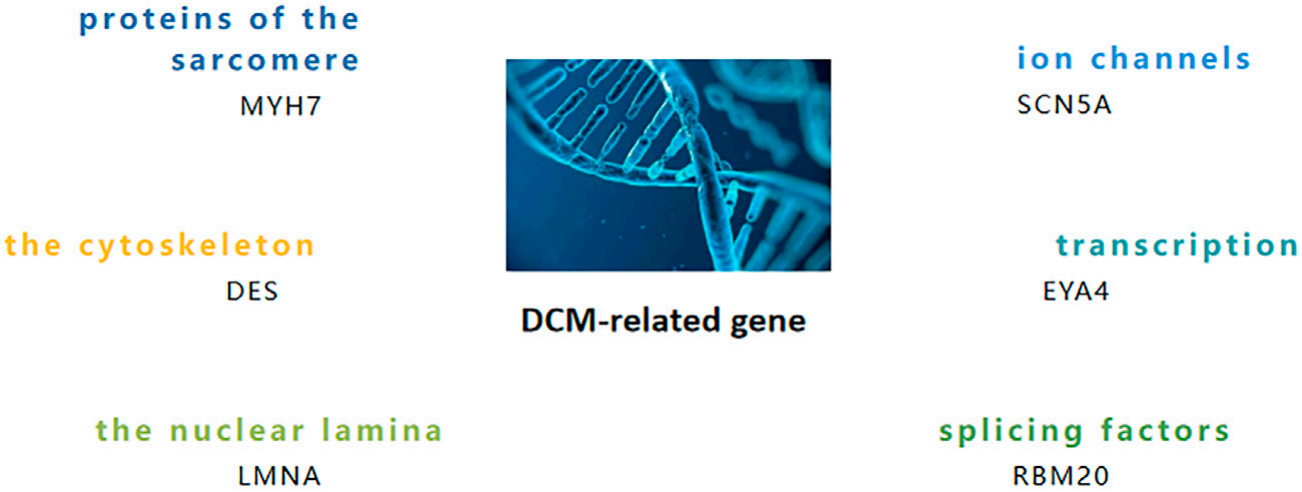
Role of the DCM-related gene.

TTN truncation variants are the most common cause in DCM patients, accounting for about 20–25% of disease cases, and have the strongest causal effect with DCM3 (Gerull et al., 2002; Herman et al., 2012). The second most common cause is mutation of the LMNA gene, which accounts for about 10% of disease cases. It is worth noting that different gene mutations can cause different phenotypes of DCM, such as arrhythmic DCM (aDCM) and non-arrhythmic DCM (naDCM). In addition, the pathogenic mechanisms of some significant gene mutations which cause DCM are summarized in Figure 1 (Fatkin et al., 1999; Li et al., 1999; Brauch et al., 2009; Herman et al., 2012). Mutations in RBM20 result in aggressive early release patterns, manifested by progressive dilation and dysfunction of the left ventricle (Hey et al., 2019). It has been found that nearly 80 different gene mutations are closely related to DCM disease, such as CAVIN4 mutations and δ-SG gene mutations.

#### The Regulationship Between DCM-Related circRNAs and Their Parental Gene

Previous circRNA profiles show 826 back-splice junctions in human left ventricle samples selected from hypertrophic or dilated cardiomyopathy patients, in which 80 junctions come from the titin gene transcript. TTN produces a class of circular RNAs that are dependent on RBM20, which has abundant titin reverse splice junction in introns flanking. These RBM20-dependent TTN circRNAs exclusively come from a region in the TTN transcript. Tijsen et al. found that selective loss of circ-TTN1 in hiPSC-CM leads to structural abnormalities in engineered heart tissues, cell apoptosis, and reduced contractility. Consistent with its SRSF10 binding, the loss of circ-TTN1 leads to abnormal splicing of important cardiomyocyte SRSF10 targets (such as MEF2A and CASQ2). Surprisingly, the loss of circ-TTN1 causes abnormal splicing of TTN itself (Tijsen et al., 2021).

It was compelling that circRNAs produced by titin mostly is involved in the development of heart diseases (Khan et al., 2016). Many circRNAs generated from titin have very complicated exon structures. According to the general situation, TTN circRNA generation comes from alternative splicing, while the more exons are circularized, and the less linearly mRNAs are produced (Ashwal-Fluss et al., 2014; Kelly et al., 2015). Interestingly, there is no TTN I band circRNA expression in the hearts of RBM20 knockout mice and human RBM20 mutation carriers. The corresponding exons are included in a large number of linear TTN transcripts. Meanwhile, this suggests a mechanism in which exons spliced from TTN pre-mRNA can be used as substrates to produce circRNAs (Khan et al., 2016). In addition to this mechanism, hundreds of expression levels of circRNAs are not affected by the host gene expression level, and some host genes can independently regulate their circRNAs (Siede et al., 2017). The same host gene has different exon splicing, which can produce circRNA variants in different cells (Hu et al., 2019).

### circRNAs in the DCM Study

#### circRNAs in the DCM Patient Heart

After digesting linear RNA with the RNase R, a large number of circular RNAs (circRNAs) are identified by RNA sequencing *via* a high-throughput sequencing platform in DCM patients (Table 1).

**TABLE 1:** Identification and evaluation of known circRNAs in DCM patient hearts.

CircRNA	Expression in DCM	Study model	Mechanism or potential application
CircRNA (CAMK2D) Khan et al. (2016)	Down	Patient’s heart	Their expression is related to RBM20 mRNA levels.
CircRNA (LAMA2) Khan et al. (2016)	Up	Patient’s heart	
CircSLC8A1 Siede et al. (2017)	Up	Patient’s heart	
CircCHD7 Siede et al. (2017)	Up	Patient’s heart	Interact with either the ribosome or Argonaute2 protein complexes.
CircATXN10 Siede et al. (2017)	Up	Patient’s heart	
CircDNA6JC Siede et al. (2017)	Down	Patient’s heart	
SCAF8_e4:TIAM2_e1 Dong et al. (2020)	Down	Patient’s heart	A theoretical basis for future studies of circRNAs in DCM.
SCAF8_e4:TIAM2_e2 Dong et al. (2020)	Down	Patient's heart	
CircFBLN1_5 Dong et al. (2020)	Up	Patient’s heart	
CircNLGN1_1 Dong et al. (2020)	Down	Patient’s heart	
CircABCC1_9 Dong et al. (2020)	Up	Patient’s heart	
CircHERC4_11 Dong et al. (2020)	Down	Patient’s heart	
CircTTN_34, 52,70,132 Dong et al. (2020)	Down	Patient's heart	
CircRYR2_71,95 Dong et al. (2020)	Down	Patient’s heart	
Has_circ_0067735 Sun et al. (2020)	Down	Child patient’s heart	Serve as non-invasive diagnostic biomarkers.
Has_circ_0070186 Sun et al. (2020)	Up	Child patient’s heart	
Has_circ_0069972 Sun et al. (2020)	Down	Child patient’s heart	
Chr7:8257935−8275635− Lin et al. (2021)	Up	Patient’s heart	A theoretical basis for future studies of circRNAs in DCM.
Chr4:187627717−187630999− Lin et al. (2021)	Up	Patient’s heart	
Chr1:219352489−219385095+ Lin et al. (2021)	Up	Patient’s heart	
Chr5:158204421−158267118− Lin et al. (2021)	Down	Patient’s heart	
Chr1:247200894−247202839− Lin et al. (2021)	Down	Patient’s heart	
Chr13:35615070−35672542+ Lin et al. (2021)	Down	Patient’s heart	

Dong et al. characterized the circRNA profile landscape of the DCM adult patient’s heart and found there are 392 circRNAs consisting of 101 upregulated and 291 downregulated circRNAs (*p* < 0.05 and FC > 2), while most of the dysregulation of circRNAs are downregulated. Notably, circRNAs in DCM are initiated from heart disease–related gene loci, which include the Rt-circRNAs produced from exons of two different neighboring genes, such as Rt-circRNA from SCAF8 and TIAM2, which very likely tend to bind with heart disease–related miRNA. It led to hypothesize that circALMS1_6 could sponge with miR-133, which plays an important role in cardiac remodeling (Dong et al., 2020). Furthermore, along a clinical investigation in identifying the roles of circRNAs in cardiac systolic and diastolic function, Lin et al. constructed the circRNAs–miRNA–mRNA gene regulatory networks including 9,585 circRNAs (231 upregulated and 85 downregulated) and 22,050 mRNAs (617 upregulated and 1125 downregulated). The comprehensive dataset assessed that the downregulated mRNA would inhibit cardiac systolica, and lack of some circRNAs would lead to DCM (Lin et al., 2021).

In addition, Sun et al. carried out a circRNA profile in 25 child patients screening novel non-invasive biomarkers for early PDCM diagnosis (Sun et al., 2020). A total of 1,156 circRNAs have the differential expression profile in PDCM under condition of fold change >2 and *p* < 0.05, including 257 upregulated and 899 downregulated circRNAs. Has_circ_0067735 and has_circ_0070186 target mRNA CACNA2D2 and IGF1. They are associated with DCM according to KEGG. “Response to wounding,” “inflammatory response,” and “cytokine secretion” are the most enrichment GO biological processes of PDCM-associated circRNAs (Sun et al., 2020), and this is different from DCM adult patients.

Recent studies show that circRYR2_71 and circRYR2_95 are downregulated in the DCM patient heart (Dong et al., 2020). Ji et al. explored the molecular pathways in miR-31-5p KO mice and cultured cardiomyocytes demonstrating the alleviated myocardial apoptosis *via* quaking and circular RNA Pan3 induced by doxorubicin treatment and QKI gene as a direct target of miR-31-5p (Ji et al., 2020). Research on the RNA-binding protein quaking (Qki) provides more evidences to support the role of the circRNA in dilated cardiomyopathy (Gupta et al., 2018). Qki5 overexpression attenuates dox-induced cardiotoxicity by inhibiting cardiac apoptosis. Special circRNAs back splice and Strn3, Fhod3, and titin have been regulated by Qki5. Importantly, sensitivity of heart cell lines toward DOX toxicity targets inhibition of titin-derived circRNAs (Gupta et al., 2018). This study indicates the important role of the circRNA in DCM in previous doctrine (Khan et al., 2016). Interestingly, van Heesch et al. reported 40 circRNAs, such as the famous CDR1as and circSLC8A1, were found to encode small peptides in the DCM patient hearts (van Heesch et al., 2019). It is worth noting that the parental gene type was determined by the specific function of circRNA itself. To be more specific, according to the healthy heart and DCM heart data, it is found that the parent genes of AUG circRNA are mostly involved in protein modification, such as ubiquitination and poly ubiquitination, while the parent genes of non-AUG circRNA are enriched in structures that bind to RNA (Stagsted et al., 2019).

### circRNAs in DCM iPSC-CMs

The study by Siede et al. and Tan et al. provides important evidences for the involvement of circRNAs in the hiPSC-CM model (Siede et al., 2017; Tan et al., 2017). Both hiPSC-CMs and human hearts show thousands of exclusively expressed circRNAs which were conserved (3874 and 6672, respectively). Due to the conservation, it is possible to study these circRNAs in large animal models as well as in human-derived cardiac cells. Both in heart development and stress treatment, dynamically variable of cicRNA expression is existed in the hiPSC-CM model. CircRNAs reverse shear produced from the exon transcript of six protein-coding genes, such as SLC8A1, ARID1A, FNDC3B, CACNA1D, SPHKAP, and ALPK2, which are highly enriched in hiPSC-CMs. In contrast, the expression levels of circAASS, circFIRRE, and circTMEFF1 are notably lower in hiPSC-CMs (Lei et al., 2018). Deep circRNA sequencing of cardiomyocyte development used in β-adrenergic stimulation revealed 4,518 circRNAs, of which the host gene set is enriched with chromatin modifiers and GTPase activity regulators (Siede et al., 2017). In addition to circ-CAMK2D and circ-LAMA2 which have statistical difference, circCACNA1D, circ-RYR2-1, circ-SLC8A1, circ-TNNI3K, circ-TTN1, circ-TTN2, circ-TTN3, circ-TTN4, and circ-TTN5 were found in the DCM patient heart with no significant difference (Khan et al., 2016).

Lei et al. detected some circRNAs, including circSLC8A1, circCACNA1D, circSPHKAP, and circALPK2, highlight their expressions during cardiac differentiation by human-induced pluripotent stem cells (hiPSCs) (Lei et al., 2018). The expression level of these circRNAs increases during cardiac differentiation and among them, circSLC8A1 is the highest level expression during differentiation. According to their high enrichment in hiPSC-derived cardiomyocytes, it led to infer that they have potential to serve as biomarkers of CMs. A previous study revealed differential expression levels of 226 circRNAs during the differentiation of human umbilical cord–derived mesenchymal stem cells into cardiomyocyte-like cells (Ruan et al., 2019). Cardiomyocyte differentiation from hiPSCs is accompanied by changes in gene expression. For example, TnnT2, Mef2c, and Myl7 are clearly upregulated in this differentiation process (Siede et al., 2017). Nkx2.5 and Isl1 are known to be expressed in early stages of cardiac formation. Furthermore, the pluripotent genes or cell type intermediate genes such as TBrachyury, MESP1, POU5F1 (OCT4), Nanog, and KLF4 are downregulated in the process toward cardiomyocyte conversion (Siede et al., 2017). These findings indicate that certain tissue- and stage-specific circRNAs can be used as biomarkers in the process of cardiomyocyte differentiation (Jakobi et al., 2020).

### Use of hiPSCs for the Study of circRNAs in DCM

The hiPSC-CM model with the p.S143P LMNA mutation successfully elucidated the mechanisms linking the LMNA alteration and its effect on DCM illness. Progressive arrhythmia or even severe arrhythmia occurs, which is similar to the clinical phenotype of DCM in patients with the p.S143P LMNA mutation, and this often leads to heart failure or sudden deaths. In addition, in the hiPSC-CM model, variability of pulsation rate, handling of abnormal calcium, and augment of sensitivity about pressure are the main causes of disease including atrioventricular conduction defects and ventricular systolic and diastolic dysfunction. Under the treatment of hypoxia stress, the disintegration of the sarcomere structure in myofibrils of hiPSC-CM shows an increasing trend. The construction of this model suggests that whether cells expressing P.S143P LaminA/C or other DCM-related mutants share some common DCM phenotypes and pathogenic characteristics, which is very important for finding suitable drugs for treatment (Shah et al., 2019).

**FIGURE 2:**
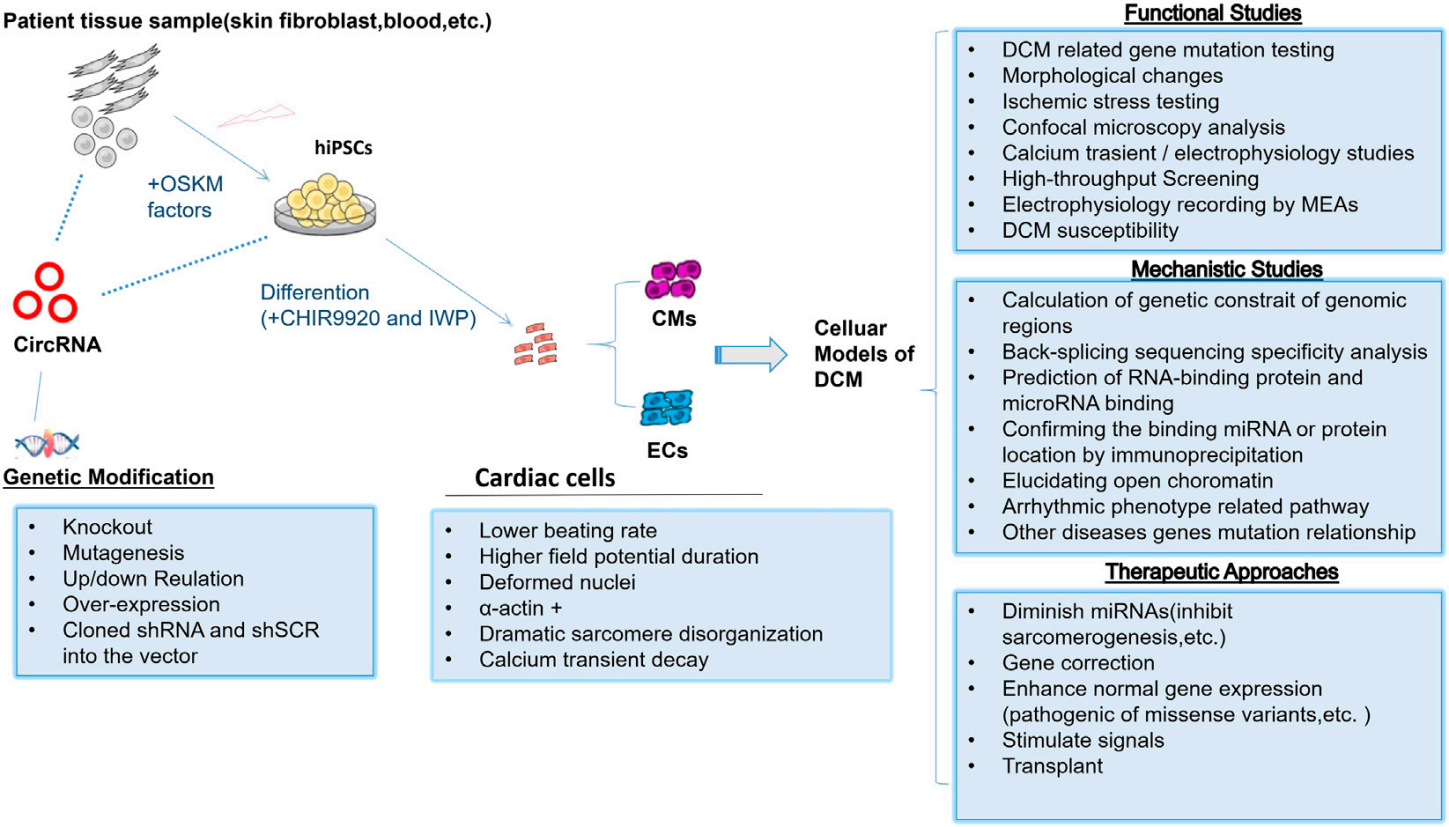
Human pluripotent stem cells as DCM models to study function and mechanism of circRNAs.

Studies with the gene knock-in animal model and/or hiPSC-CM model would get confidential evidences of the genotype–phenotype correlation, and therefore they offer a powerful tool to interpret the physiological functions of gene mutation–related heart diseases. When establishing a preliminary model of DCM-related circRNA disease, it is necessary to select a circRNA that has a weak effect on the expression of the parental gene, which is helpful to eliminate the interference if the parental gene has a function in the DCM disease progress. Also, it is a good choice to start with some circRNAs related to RBM20 expression as mentioned before. Consequently, studies of molecular mechanisms of circRNAs have just recently begun, so we note that this research in pluripotent stem cell–derived cardiomyocyte about heart diseases is still in its nascent stages, and currently the study mostly focus on its expression situation; only a few research studies explore molecular mechanisms of circRNAs in pluripotent stem cell–derived cardiomyocyte. Recently, Anke et al. successfully constructed the hiPSC-CM model with circular RNA TTN1 deletion (Tijsen et al., 2021). In this study, the most abundant RMB20-dependent circRNA in the human heart was selected, of which parental gene TTN variants are found in 20–30% patients suffering from DCM. The cTTN1 was expressed highly in healthy people but down-regulated in patients with DCM. Therefore, the researcher decided to build a downregulation circRNA model instead of an upregulation one. After inducing the selective loss of cTTN1 in hiPSC-CMs, it was found that the loss of cTTN1 can cause abnormal cardiac tissue structure, apoptosis, and reduced contractility. The cTTN1 mechanisms of action are summarized as follows: 1) The special motif AAAGAACC was found in the back splice junction of cTTN, which has a role in binding the splice regulator SRSF10. 2) The loss of cTTN1 would move most of RBM20 from the nucleus to the cytoplasm, resulting in splicing deletion of the RBM20 and SRSF10 targets. If we want to build the model, we can refer to the figure (Figure 2).

### Challenges of hiPSC Modeling and Translational Aspects

It seems that hiPSC could provide an unlimited cell source for regenerative medicine in heart transplant and card cytotoxicity experiments. However, several weaknesses, include genomic instability causing chromosomal aberrations, low frequency of iPSCs, wide variation of the quality of iPSC colonies, and hinder iPSC applications even for *in vitro* analysis (Mayshar et al., 2010; Garber, 2015).

Cardiac muscles are composed of cardiomyocytes, extracellular collagenous matrix, vasculature, and *etc*. In addition, cardiomyocytes take only a small part in 25–35% of myocardial tissue (Pinto et al., 2016). For example, iPSC-derived organoids lack enough cell maturation and precise microvascular formation. The procedures of culturing iPSC-CM similarity to ESCs are not complex, but the composition of intrinsic properties of iPSC-derived cardiomyocytes varies under pathological conditions. Therefore, it is a difficult problem to use iPSC-derived cardiomyocytes for modeling heart diseases *in vitro*, although the survival of iPSC-derived cardiomyocytes and the complexity of the model have been used to significantly improve accuracy during the past few years (Breckwoldt et al., 2017; Lemoine et al., 2017; Weinberger et al., 2017; Castro et al., 2019; Meyer et al., 2019).

Uncertainties in cytogenetics and epigenetics make it difficult to directly correlate *in vitro* functional effects and specific genetic variations if using patient-derived iPSCs (Dzilic et al., 2018). In addition, gene variant mutations might be produced and even accumulate during the long time of culturing patient-derived iPSCs (Liu et al., 2014; Yoshihara et al., 2017). Short tandem repeat (STR) analysis is always used for the genetic stability test, and therefore using early passages is necessary.

In the early days, hiPSC-CM treatment was used to fill missing tissue induced by MI. The induced pluripotent stem cells were considered to be superior to human embryonic stem cells (ESCs) for repairing damaged myocardium (Liao et al., 2019). But hESC-VCs could alleviate a severely abnormal heterogeneity of myocardial bioenergetics in hearts (Xiong et al., 2012). Due to the low transplant survival rate and other reasons, trying for over-expression of angiopoietin-1 (Ang-1) or add thymosinβ4 (Tb4) could improve engraftment and reparative potency of transplanted hiPSC-CMs in a porcine model (Tan et al., 2021; Tao et al., 2021). Finally, pluripotent stem cell (PSC)–derived cardiomyocytes (hiPSC-CMs) were proven to enhance cardiac function by integrating into infarcted hearts in the porcine model of myocardial infarction (Ye et al., 2014).

hiPSCs were believed to be more readily available for cellular transplantation and personalized therapies (Su et al., 2021). The stable CM graft formation in the rodent infarcted hearts after transplanted hiPSC-CMs or hiPSC-CVPCs was an encouraging progress in technology. Overall, the systematic development of human cardiac organoids would accelerate cardiac drug discovery and personalize cardiac treatment in the future. Therefore, iPSC-derived cardiomyocytes should also be linked to animal models or the study of complicated explanted human myocardial tissue in order to obtain actual clinical benefit.

## Discussion

Although significant efforts have been made in genetic variants–induced pathophysiological changes for human heart diseases such as DCM, HCM, and various types of long QT syndrome (LQTS), our understanding of circRNA function in heart disease is still very limited. In recent years, high-throughput sequencing detection technologies continue to surmount, which further have improved our knowledge about epigenetic contribution to pathogenesis of heart disease. Increasing evidence supports circRNAs could regulate cardiac hypertrophy, heart failure, and myocardial fibrosis *via* regulation signaling pathways or sponging with some miRNAs. CircRNA mm9_circ_012559, which downregulated, can target with miR-223 to aggravate heart hypertrophy (Wang et al., 2016). CircRNA (HRCR) contains a group of 36 circRNAs which were all upregulated in the heart. HRCR decreases the level of ARC expression and enhances myocardial hypertrophy produced by isoproterenol (ISO) *via* sponging with downregulated MiR-223 (Wang et al., 2016). CircSlc8a1 can adsorb miR-133a which has an important role in cardiac hypertrophy in cardiomyocytes. Therefore, circSlc8a1 knockdown weakens cardiac hypertrophy from excessive pressure (Lim et al., 2019). The overexpression of circRNA_000203 enhances cell size by promoting atrial natriuretic peptide and β-myosin heavy chain expression in neonatal mouse ventricular cardiomyocytes. The upregulated circRNA_000203 in Ang-II–infused mice enhances cardiac hypertrophy and acts with its target siRNA to inhibit hypertrophy in turn (Nishi et al., 2010). However, the exact mechanisms of how these circRNAs affect the progression of DCM remains largely unknown. Some circRNAs are differentially expressed and detected easily in DCM diseases and could play an important role in other heart diseases. For example, circSlc8a1 has also upregulated in myocardial infarction, and it was confirmed as auxiliary diagnostic markers for SCD caused by acute IHD (Tian et al., 2021). This poses a great challenge for identification of DCM-related circRNAs as specific heart disease molecular markers when facing complicated and combined heart disease. More importantly, the ceRNA theory has been the main hypothesis of how circRNAs function as miRNA sponges in heart diseases. However, it is skeptical about whether the physiological expression level of a single circRNA is sufficient to absorb its target miRNAs because miRNA targets including lncRNAs, cirRNAs, mRNAs, and pseudogenes and the efficient regulation are finally determined by the number of common miRNAs and the target binding sites (Le et al., 2017). It is essential for the future study to investigate all the genes composed of the ceRNA network for a better understanding of DCM, especially mostly miRNAs-specific expression only in a certain disease. At last, the detection method of the circRNA has been upgraded from the first- and second-generation sequencing technologies to nanopore third-generation sequencing technologies, but most of the research studies about circRNAs on DCM disease are based on the first- and second-generation sequencing technology. Many circRNAs with full length> 500 nt and specific variable shear excision events cannot be detected by the first- and next-generation sequencing, which may lead to missed detection of some circRNAs that play an important role in the occurrence and development of DCM disease (Rahimi et al., 2021).

As mentioned previously, most of circRNA studies are conducted in mouse models, failing to mimic the *in vivo* patients. Therefore, hiPSC from patients with various heart diseases should be used as a more relevant physiological model. However, it is worthy of noting that using hiPSC as a disease model faces some challenges as follows: In a hiPSC-CM model, cLQTS2 and Kv11.1 activators could restore normal heart signaling, but at the same time there may be a hazard of overcorrection that reduces itself being pro-arrhythmic (Perry et al., 2020). HiPSC-CM do not express other components that make up the protein of cardiomyocytes, including key Ca2+ processing components and contractile elements, despite it could remedy arrhythmic Ca2+ transients and alleviates declined Ca2+ transport in a DCM model; this leads to limited observations (Stroik et al., 2020). But the advantage is always obvious too. The *in vitro* phenotype of hiPSC-CM of asymptomatic and symptomatic individuals with LQT2 differs in the level of CM aggregation; an increase in arrhythmia is observed in symptomatic hiPSC-CM, which makes it convenient for us to use hiPSC-CM to observe the different phenotypes of mutation carriers with different clinical phenotypes (Shah et al., 2020).

The latest research about using hiPSCs for the study of circRNAs in DCM declares that if there is no RBM20 mutation detected when the phenotype resembles the phenotype of DCM with arrhythmias as observed in RBM20 mutation carriers, it is necessary to consider variants in the I-band region (Tijsen et al., 2021) because hindering formation or function of these TTN circular RNAs which stem from the I-band region also may have the DCM clinical phenotype. This will bring some new perspectives to gene-targeted therapy, for example, some gene mutation or deletion diseases that are only judged from clinical symptoms. When the targeted gene therapy is ineffective according to clinical symptoms which are reduced by DCM-related gene mutations or deletion, factors caused by non-coding RNA disorders such as circRNA could be considered. The current research has always emphasized on downregulated circRNAs in DCM patients and hiPSCs-CM. However, whether the upregulated circRNAs in the DCM patient’s heart and hiPSCs-CM have a similar mechanism or a different mechanism could only be further studied.

The short hairpin RNA (shRNA) and antisense oligonucleotide (ASO) have been considered to target mRNAs in the hiPSC-CM model to ameliorate phenotypes of disease. Importantly, Shah, D. gave an outstanding article about these two methods of silencing as therapeutic treatment for MYH7 gene mutation cardiomyopathy. In addition, they found shRNA silencing way may prove to be more efficacious toward ASO silencing in the treatment of the human HCM model (Dainis et al., 2020).

To sum up, there are several examples of human DCM diseases related to titin gene and LMNA gene which have been successfully modeled by linking the homologous cells in 3D fabricated tissue culture models. circRNAs are potential candidates as biomarkers for the diagnosis of DCM. Moreover, most circRNAs have sequence conservation between mammals, providing favorable conditions for the study in cells or animal disease models. hiPSC provides a much facile and accessible way to obtain human cells, while the new differentiation method can cultivate a large number of different classes of cardiomyocytes. Our review provides a new perspective of iPSC-derived atrial cardiomyocytes for exploring the role of circRNAs in the pathophysiology of DCM and offers a platform for evaluating potential treatment methods.
